# Comparative Transcriptome Analysis Revealed Two Alternative Splicing bHLHs Account for Flower Color Alteration in Chrysanthemum

**DOI:** 10.3390/ijms222312769

**Published:** 2021-11-25

**Authors:** Lili Xiang, Xiaofen Liu, Yanna Shi, Yajing Li, Weidong Li, Fang Li, Kunsong Chen

**Affiliations:** 1 College of Agriculture & Biotechnology, Zijingang Campus, Zhejiang University, Hangzhou 310058, China; 11516055@zju.edu.cn (L.X.); lmqlxf119@163.com (X.L.); shiyanna@zju.edu.cn (Y.S.); liyajing_0526@163.com (Y.L.); akun@zju.edu.cn (K.C.); 2 Zhejiang Provincial Key Laboratory of Horticultural Plant Integrative Biology, Zijingang Campus, Zhejiang University, Hangzhou 310058, China; 3 The State Agriculture Ministry Laboratory of Horticultural Plant Growth, Development and Quality Improvement, Zijingang Campus, Zhejiang University, Hangzhou 310058, China; 4 Hunan Horticultural Research Institute, Changsha 410125, China; huangguolin2002@126.com; 5 Hunan Key Laboratory of Germplasm Innovation and Comprehensive Utilization of Landscape Flowers, Changsha 410125, China

**Keywords:** MYB, bHLH, anthocyanin, alternative splicing, chrysanthemum

## Abstract

‘Jimba’ is a white chrysanthemum cultivar, which occasionally and spontaneously produces red flower petals under natural cultivation due to cyanidin-based anthocyanin accumulation. To investigate the underlying mechanism of this process, a comparative transcriptome was analyzed between white and turning red ‘Jimba’. The structural and regulatory genes of anthocyanin pathway were significantly up-regulated in turning red ‘Jimba’. Among them, two alternative splicings, *CmbHLH2* and *CmbHLH2.1*, showed the most significantly up-regulated in turning red tissue. Transiently over-expressed *35S::CmMYB6-CmbHLH2* strongly induced anthocyanin accumulation in ‘Jimba’ flower petals, while moderate amount of anthocyanin was detected when over-expressed *35S::CmMYB6-CmbHLH2.1*. Both CmbHLH2 and CmbHLH2.1 could interact with CmMYB6 to activate *CmDFR* promoter according to Yeast two-hybrid and dual-luciferase assay. Moreover, CmMYB6-CmbHLH2 but not CmMYB6-CmbHLH2.1 could activate the *CmbHLH2* promoter to provide positive feedback loop regulation. Taken together, it suggested that both CmbHLH2 and CmbHLH2.1 involved in regulation flower color alteration in turning red ‘Jimba’, and CmbHLH2 played a predominant role in this process.

## 1. Introduction

Chrysanthemum (*Chrysanthemum* × *morifolium* Ramat) is one of the most popular cut flowers which distribute a wide range of colors [1,2]. Field investigation showed that 202 out of 317 chrysanthemum cultivars flower color tended to turn red, which was particularly common in white chrysanthemum cultivars [3]. ‘Jimba’ is a popular white chrysanthemum cultivar, which only produced flavonoids instead of anthocyanins [4]. However, sometimes, the flower color of ‘Jimba’ turns red, which occurs spontaneously under natural cultivation due to the accumulation of cyanidin-based anthocyanins [2].

Anthocyanins are products of a series of enzymatic steps, including the early biosynthetic genes (*CHS*, *CHI*, *F3H* and *F3′H*), late biosynthetic genes (*DFR*, *ANS* and *UFGT*) and transfer genes (*GST*), which are conservatively regulated by the MYB-bHLH-WD40 (MBW) complex [5,6,7]. Among them, MYB is the most specialized in terms of spatial expression, timing of expression and target genes [8]. By contrast, the anthocyanin-related bHLHs play more pleiotropic roles, being shared between two or more cellular processes [9]. The interaction with MYB is essential for the ability of bHLHs to activate all known flavonoid biosynthetic genes, resulting in anthocyanin accumulation [10,11]. Anthocyanin-related bHLHs belong to the IIIf subfamily, which is recognized as a MIR (MYB interaction) domain. Moreover, the C-terminal region mediated homo-/heterodimers formation was reported necessary for regulatory effects [11,12,13]. 

Generally, there are several MYBs and bHLHs which are redundantly responsible for anthocyanin regulation. These redundant regulators might be paralogous genes, alleles or alternative splicings. For example, the alternative splicing of R2R3, MYB affects anthocyanin biosynthesis in tomato fruits [14]. In *Arabidopsis*, two MYB activators (PAP1 and PAP2), three bHLH activators (TT8, EGL3 and GL3) and at least two R3 repressors (MYBL2 and CPC) can physically interact with each other to form various transcriptional complexes together with WD-repeat protein (TTG1) [6,15,16]. The expression changes or loss-function mutant of these genes always lead to the alteration of anthocyanin contents, resulting in color alteration consequently [17,18,19,20,21]. 

In chrysanthemum, several anthocyanin-related MYBs and bHLHs have been identified in previous studies, including the light-response R2R3-MYB *CmMYB4*, *CmMYB5*, *CmMYB6* and *CmMYB7*, which could activate or inhibit anthocyanin accumulation when ectopic over-expressed in tobacco [22,23]. CmbHLH2 and CmMYB6 could form a complex to activate anthocyanin pathway genes, while their regulatory effect could be inhibited by CmMYB#7, a passive anthocyanin repressor, through hindering the formation of activation MYB-bHLH complex [2,24,25].

The flower color alteration in white ‘Jimba’ is an undesirable commodity trait for market. However, the underlying mechanism of this process was not fully understood. Here, a comparative transcriptome was analyzed between white and turning red ‘Jimba’, and the differentially expressed transcription factors were screened out. Among them, two alternative splicing bHLHs showed the most significantly up-regulated. The gene structure, gene functions and regulation mechanisms of two bHLHs were further analyzed. Furthermore, the hierarchical and feedback mechanisms among anthocyanin regulators in chrysanthemum were analyzed and discussed.

## 2. Results

### 2.1. Anthocyanin Contents in White and Turning Red ‘Jimba’

**Figure 1:**
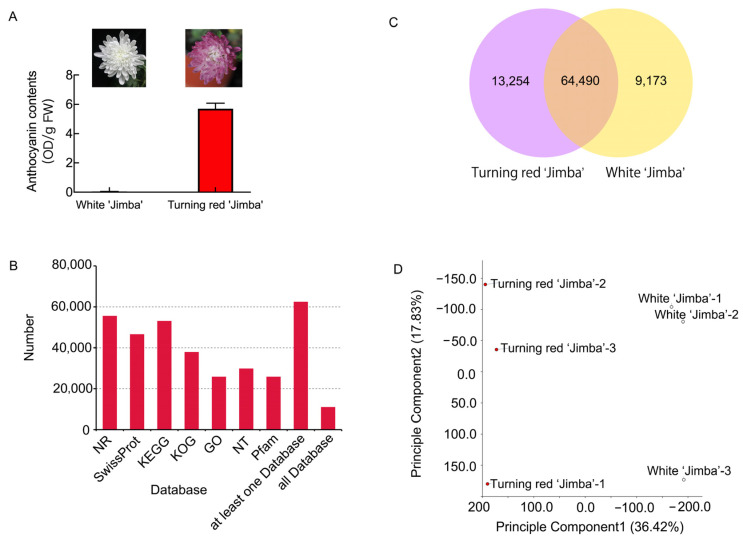
Transcriptome analysis of white and turning red ‘Jimba’. (**A**) Flowers and anthocyanin content of white ‘Jimba’ and turning red ‘Jimba’ at full bloom stage. (**B**) Functional annotation of transcriptome data using NR, SwissProt, KEGG, KOG, GO, NT and Pfam database. (**C**) Venn diagram of expressed unigenes in each sample. (**D**) Principal component analysis of gene expression in each library.

‘Jimba’ is a white chrysanthemum cultivar, but it occasionally and spontaneously produces red flower petals under natural cultivation, which is attribute to cyanidin-based anthocyanin accumulation based on previous study [2]. As shown in Figure 1A, the anthocyanin contents in white ‘Jimba’ and turning red ‘Jimba’ were 0.04 OD/g FW and 5.70 OD/g FW, respectively.

### 2.2. Transcriptome Analysis of White and Turning Red ‘Jimba’

A comparative RNA sequencing was carried out to investigate the underlying mechanism. Flower petals of full-bloom stage were used for cDNA library construction. A total of 38.44 Gb filtered clean data was produced with an average of 6.4 Gb clean reads for each sample. The percentage of Q30 in each sample was not less than 94%, and the GC content was about 42–45%. After assembling the clean reads, a total of 73,663 and 77,744 unigenes were obtained from the white and turning red ‘Jimba’, respectively. Among them, 10,906 unigenes were annotated in all databases and 62,382 unigenes were annotated in at least one database (Figure 1B). Furthermore, Venn plots and PCA score of gene expression among two samples revealed the effectiveness of the data (Figure 1C,D).

### 2.3. Differentially Expressed Genes in White ‘Jimba’ and Turning Red ‘Jimba’

**Figure 2:**
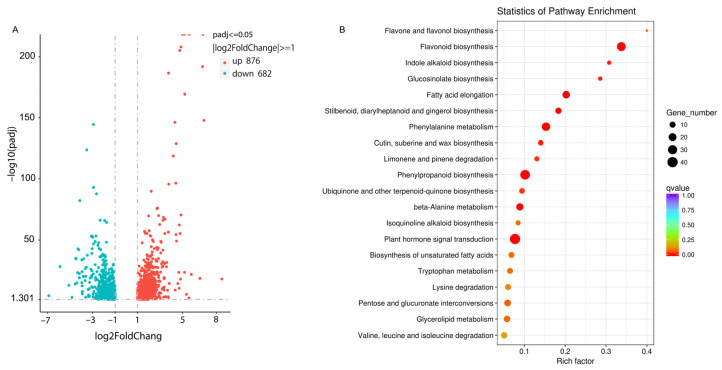
Volcano plot and KEGG pathway enrichment of DEGs. (**A**) Volcano plot and (**B**) KEGG pathway enrichment of DEGs in turning red ‘Jimba’ compared to white ‘Jimba’.

Using padj ≤ 0.05, |Log_2_Fold change| ≥ 1, and FPKM ≥ 10 as cut-off, a total of 1558 DEGs (differentially expressed genes) were obtained; among them, 876 were up-regulated and 682 were down-regulated in turning red ‘Jimba’ (Figure 2A). 

Based on KEGG enrichment analysis, the differentially expressed genes were the most significantly enriched in the flavonoid biosynthesis pathway (Figure 2B), where 27 transcripts were differentially expressed with a background of 80 transcripts annotated to flavonoid pathway in KEGG database. 27 transcripts were redundantly annotated as C4H (KO ID: K00487), CHS (KO ID: K00660), CHI (KO ID: K01859), F3H (KO ID: K00475), F3′H (KO ID: K05280), DFR (KO ID: K13082), ANS (KO ID: 05277) and CCoAOMT (KO ID: 00588). Among them, 25 were up-regulated and 2 were down-regulated.

### 2.4. Differentially Expressed Genes of Anthocyanin Pathway

**Figure 3:**
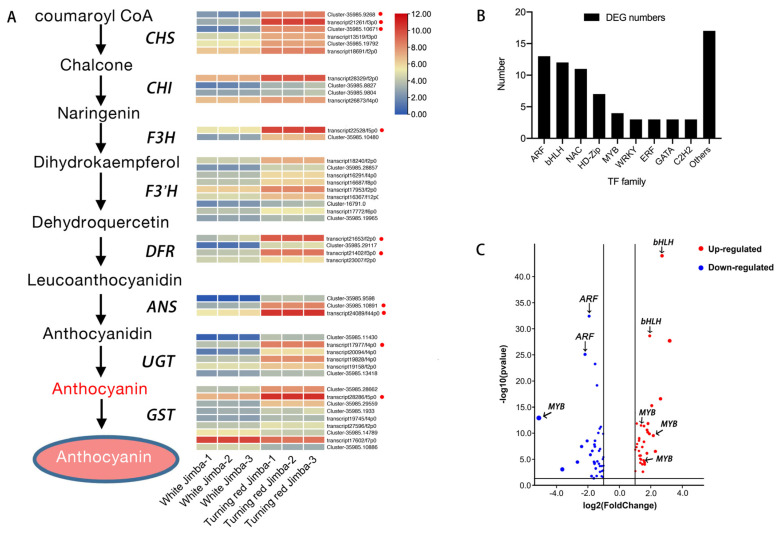
DEGs of anthocyanin pathway genes and transcription factors. (**A**) Anthocyanin pathway genes and heatmap of anthocyanin pathway genes in white and turning red ‘Jimba’. Gradient colors represent log_2_ FPKM in gene expression, while color from blue to red represents the expression levels from low to high. The top 10 transcripts with the most significant change were designated with red solid circles. (**B**) Numbers of differentially expressed transcription factors in each family. (**C**) Volcano plot of differentially expressed transcription factors. DEGs of *MYBs* and *bHLHs* and the most significantly changed TFs were marked with arrows.

Anthocyanin biosynthetic pathway is a branch of the flavonoid pathway. Thus, the DEGs of anthocyanin pathway were further screened out based on the annotation, including early biosynthetic genes (*CHS*, *CHI*, *F3H* and *F3′H*), late biosynthetic genes (*DFR*, *ANS* and *UGT*) and anthocyanin transfer genes *GST* (glutathione S-transferase). Worthy to mention is that all biosynthetic genes have at least one paralogous, and were significantly up-regulated in turning red ‘Jimba’ compared to white ‘Jimba’ (Figure 3A). Fold change of the highest expressed paralogous of *CHS*, *CHI*, *F3H*, *F3′H*, *DFR*, *ANS*, *UGT* and *GST* were up to 113-, 7-, 31-, 5-, 49-, 36-, 27- and 13-fold respectively.

### 2.5. Differentially Expressed Transcription Factors

TFs play an important role in anthocyanin biosynthesis, so we checked differentially expressed TFs in our samples. A total of 1973 unigenes were predicted to be transcription factors (TFs) by iTAK software. Among them, 76 were differentially expressed, which distributed across 18 TF families including *ARF* (13 members, 17.1%), *bHLH* (12 members, 15.8%), *NAC* (11 members, 14.5%), *HD-Zip* (7 members, 9.2%) and *MYB* (4 members, 5.2%) (Figure 3B). 

Among these differentially expressed TFs, two bHLHs showed the most significantly up-regulated, up to 6.55- and 3.81-fold, respectively, and two ARFs showed the most significantly down-regulated (Figure 3C). 

### 2.6. Phylogenetic Analysis of Differentially Expressed MYBs and bHLHs

**Figure 4:**
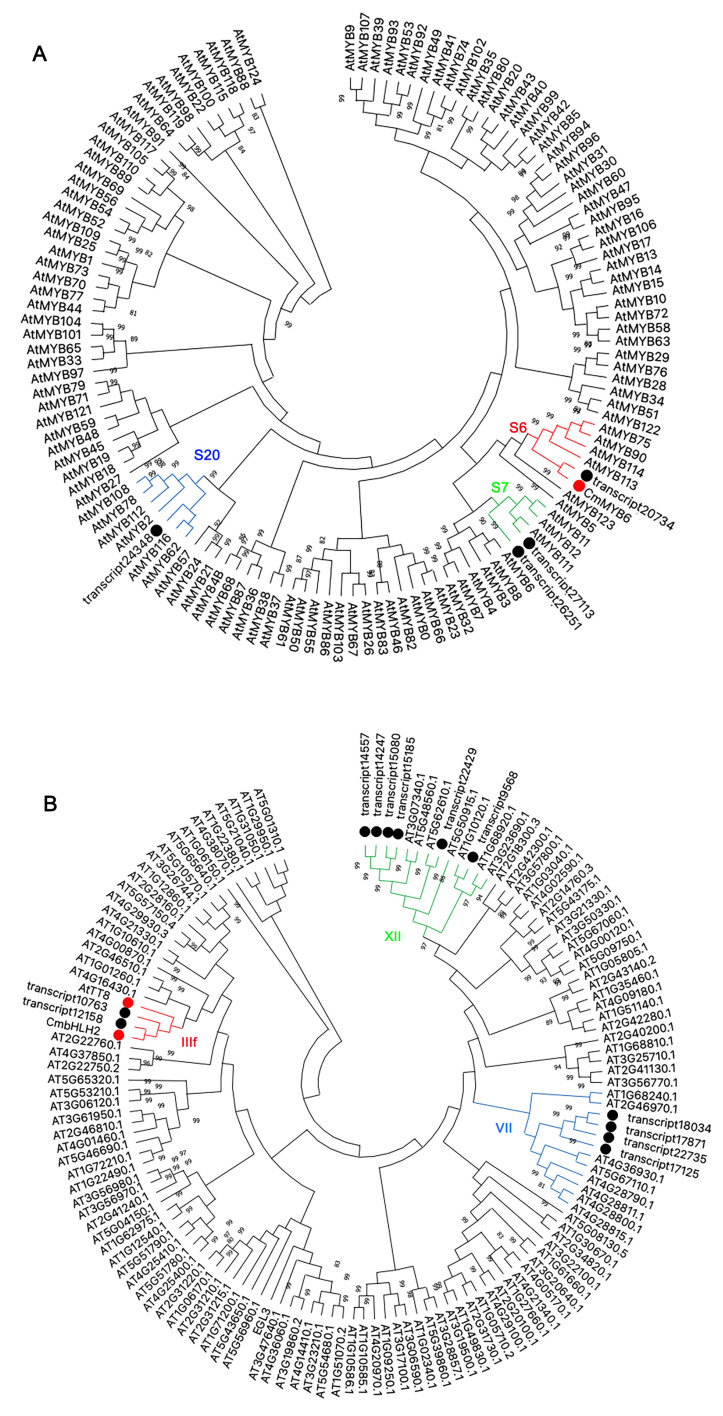
Phylogenetic analysis of CmMYBs and CmbHLHs together with MYBs and bHLHs of *Arabidopsis*, respectively. (**A**) CmMYBs were analyzed together with 126 MYBs from *Arabidopsis*, 4 differentially expressed MYBs were marked with a black solid circle. Reported anthocyanin regulators were marked with a red solid circle. (**B**) CmbHLHs were analyzed together with 120 bHLHs from *Arabidopsis*, 12 differentially expressed bHLHs were marked with a black solid circle. Reported anthocyanin regulators were marked with a red solid circle. Neighbor-joining trees were constructed with 1000 replications of bootstrap.

Since MYB and bHLH are important regulators for anthocyanin pathway, the DEGs of 12 bHLHs and 4 MYBs were further analyzed. Phylogenetic tree was constructed using 4 MYBs together with 126 MYBs from *Arabidopsis* (Figure 4A). Four MYBs were separated into three clades. One of them (transcript20734) was clustered into the S6 subfamily and showed a close relationship with anthocyanin activators, including AtMYB75 (PAP1), AtMYB90 (PAP2) and CmMYB6. Two MYBs (transcript26251 and transcript27113) were clustered into the S7 subfamily and showed a close relationship with AtMYB11, AtMYB12 and AtMYB111, which related to the flavonoid pathway, while the other one (transcript24348) was clustered into the S20 subfamily. 

The evolutionary relationship of 12 bHLHs together with 120 bHLHs from *Arabidopsis* was also analyzed by phylogenetic tree (Figure 4B). Twelve bHLHs were clustered into three subfamilies. Two of them (transcript12158 and transcript10763) were clustered into the IIIf clade, and showed a close relationship with anthocyanin activators of CmbHLH2 and TT8. Six bHLHs (including transcript14557, transcript14247, transcript15080, transcript15185, transcript22429 and transcript9568) were clustered into the XII clade and showed a close relationship with CIB1-4, which responds to blue light and regulate flowering [26]. The others (including transcript18034, transcript17871, transcript22735 and transcript17125) were clustered into the VII clade, which responds to environmental stimuli [27].

### 2.7. Full-Length CDS Clone and Sequence Alignment of Anthocyanin-Related MYBs and bHLHs

Based on the phylogenetic analysis, one MYB (transcript20734) and two bHLHs (transcript12158 and transcript10763) were predicted to be anthocyanin activators. The transcript20734 encoded a MYB protein with 254 amino acids, and shared 97% identities with reported anthocyanin activators CmMYB6 (genbank: AKP06190.1). The transcript12158 and transcript10763 encoded bHLH proteins with 613 and 614 amino acids, respectively, and shared 96% and 92% identities with previous CmbHLH2 (genbank: ALR72603.1). The differences between two bHLHs were 26 amino acids at the C-terminal. 

**Figure 5:**
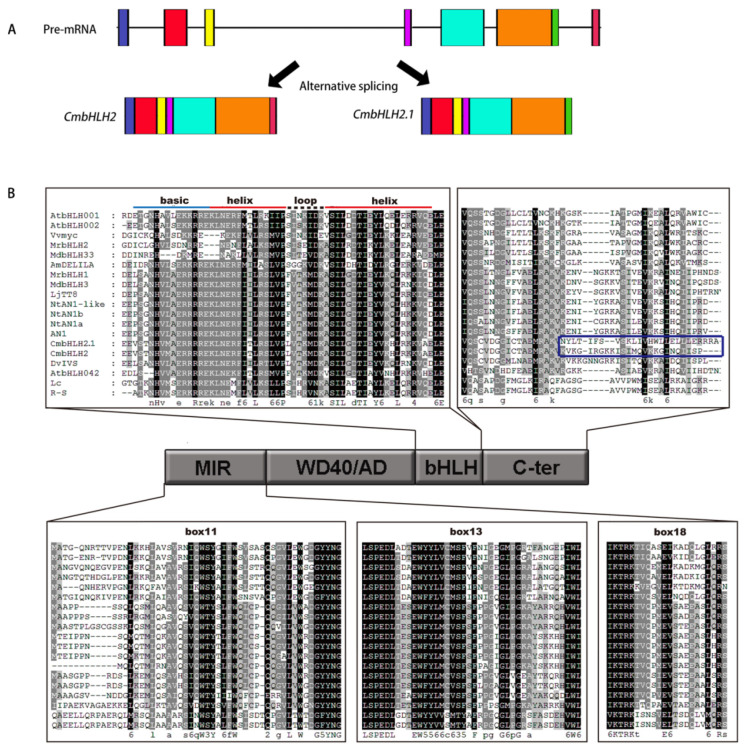
Structure diagram and protein sequence alignment of two bHLHs. (**A**) Structure diagram of two alternative splicings of *bHLH2* Pre-mRNA. Rectangles with different colors indicate exons. (**B**) Protein sequence alignment of two bHLHs together with IIIf bHLHs from other species. The conserved basic helix-loop-helix domain was marked with red and blue lines. The MIR (MYB interact region) includes box11, box13 and box18. The sequence differences between two CmbHLHs were marked with a blue box.

Further analysis found that they were mapped on the same gene (Gene ID: Cse_sc003162.1_g080.1) in the chrysanthemum genome (http://mum-garden.kazusa.or.jp accessed on 15 November 2021), and the gene structure analysis indicated that the difference in the C-terminal is due to the variable splicing of the 6th introns of *bHLH2* (Figure 5A). Thus, the other one was designated as *CmbHLH2.1*. Furthermore, full-length CDS of MYB and bHLHs were cloned from both white and turning red ‘Jimba’ and protein sequence alignment revealed all of them to have no differences between two samples.

Protein sequence alignment together with characterized bHLHs from other species indicated that two bHLHs both have a conserved basic helix-loop-helix (bHLH), which accounts for DNA binding, and an MIR domain (including box11, box13 and box18) accounts for the MYB interaction (Figure 5B). 

### 2.8. Analysis of the Protein–Protein Interaction between Regulators and the Protein–DNA Binding Activity of bHLHs on the CmDFR Promoter

**Figure 6:**
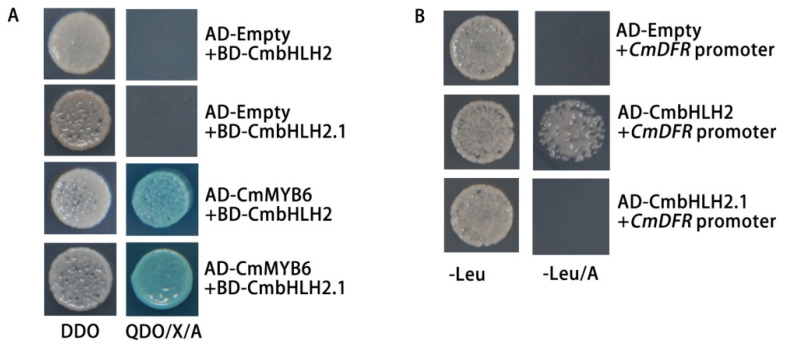
Protein–protein and protein–DNA interaction in yeast. (**A**) Protein–protein interaction between CmbHLHs and CmMYB6. (**B**) Interaction of CmbHLH2 and CmbHLH2.1 with the *CmDFR* promoter. DDO, SD/−Leu/−Trp; QDO, SD/−Ade/−His/−Leu/−Trp; X, X-α-Gal; A, aureobasidin A; AD, GAL4 activation domain; BD, GAL4 DNA binding domain.

The protein–protein interactions of CmbHLH2 and CmbHLH2.1 together with CmMYB6 were analyzed using the Yeast two-hybrid. The Y2H strain co-transformed CmMYB6-AD together with bHLH2.1-BD or CmbHLH2.1-BD; both could grow on a QDO (−His/−Ade/−Trp/−Leu) plate containing AbA (aureobasidin A) (Figure 6A), indicating that both of them could physically interact with CmMYB6.

Yeast one-hybrid assays were conducted to test whether two bHLHs could bind to the *CmDFR* promoter. When transformed bHLH2-BD into Y1H strain containing *CmDFR* promoter, robust yeast could grow on the SD/−Leu plate with AbA. However, when transformed with bHLH2.1-BD, no yeast could be growing on the SD/−Leu plate with AbA (Figure 6B), indicating that CmbHLH2 could bind to the *CmDFR* promoter but not CmbHLH2.1. 

### 2.9. Transiently Over-Expressed 35S::CmMYB6-CmbHLH2 and 35S::CmMYB6-CmbHLH2.1 in White ‘Jimba’

**Figure 7:**
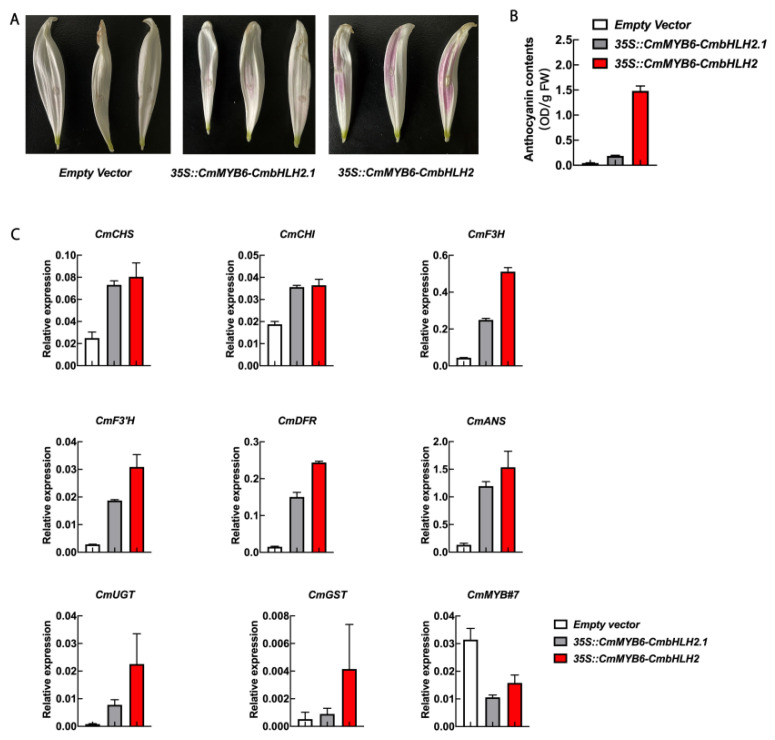
Transiently over-expressed *CmMYB6* together with *CmbHLH2* and *CmbHLH2.1* in white ‘Jimba’. (**A**) The flower petals infiltrated with Empty vectors, *35S::CmMYB6-CmbHLH2* and *35S::CmMYB6-CmbHLH2.1*. (**B**) Relative anthocyanin contents of transformed patches. (**C**) Relative expression of anthocyanin structural genes and regulators. Error bar indicated the SE of three independent biological replicates.

Transiently over-expressed assay in white ‘Jimba’ flower petals were carried out to investigate the function of *CmbHLH2* and *CmbHLH2.1*. No anthocyanin could be detected when infiltrated with *35S::CmMYB6*, *35S::CmbHLH2* or *35S::CmbHLH2.1* separately (data not shown). When *35S::CmMYB6-CmbHLH2* is over-expressed, flower petals began turning red on the fourth day after infiltration, and only a moderate amount of anthocyanin could be observed when *35S::CmMYB6-CmbHLH2.1* was over-expressed (Figure 7A).

Relative anthocyanin contents in transiently transformed petals were detected on the eigth day after infiltration. Anthocyanin contents in transformed patches of Empty vector, *35S::CmMYB6-CmbHLH2.1* and *35S::CmMYB6-CmbHLH2* were 0.04 OD/g FW, 0.19 OD/g FW and 1.48 OD/g FW, respectively (Figure 7B).

The relative expression of anthocyanin pathway genes in transformed petals was analyzed by RT-qPCR (Figure 7C). Compared to empty vector, anthocyanin pathway genes, including *CHS*, *CHI*, *F3H*, *F3′H*, *DFR*, *ANS*, *UGT* and *GST*, were strongly up-regulated in *35S::CmMYB6-CmbHLH2*, up to 3.22-, 1.95-, 11.72-, 10.85-, 16.06-, 11.58-, 28.11- and 7.95-fold, respectively. Over-expressed *35S::CmMYB6-CmbHLH2.1* also activated all anthocyanin pathway genes with a relatively lower fold change compared to *35S::CmMYB6-CmbHLH2* (Figure 7C). 

The expression of *CmMYB#7*, a reported anthocyanin repressor, was significantly inhibited in both *35S::CmMYB6-CmbHLH2* and *35S::CmMYB6-CmbHLH2.1* transformed petals, suggesting that *35S::CmMYB6-CmbHLH2* and *35S::CmMYB6-CmbHLH2.1* induced anthocyanin synthesis both by enhancing anthocyanin biosynthetic genes and inhibiting *CmMYB#7*. 

### 2.10. Regulation Effects of CmMYB6 Together with bHLH2 and bHLH2.1 on the CmDFR Promoter

**Figure 8:**
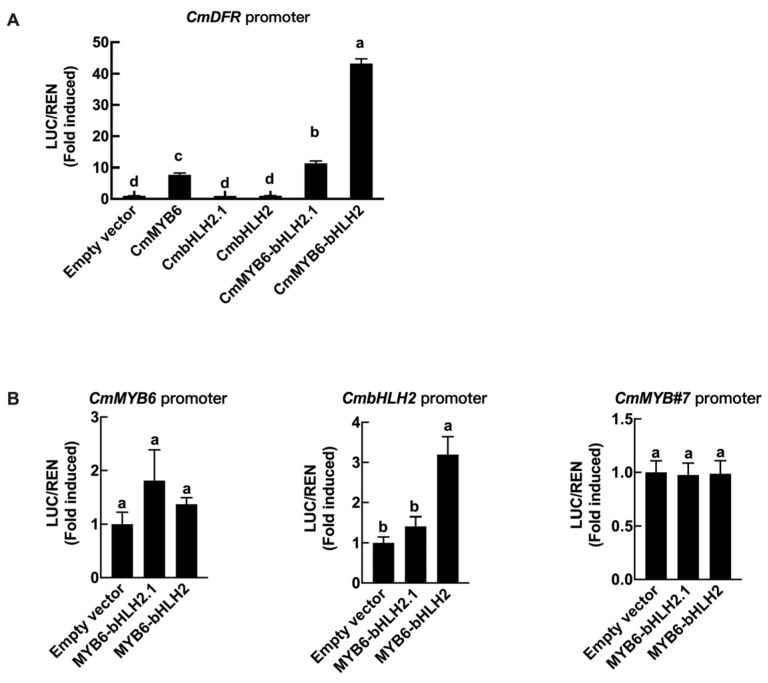
Regulatory effects of CmMYB6-CmbHLH2 and CmMYB6-bHLH2.1 on the *CmDFR*, *CmMYB6*, *CmMYB#7* and *CmbHLH2* promoters. (**A**) Regulatory effects of CmMYB6-CmbHLH2 and CmMYB6-bHLH2.1 on the *CmDFR* promoter. (**B**) The regulatory effects of CmMYB6-CmbHLH2 and CmMYB6-bHLH2.1 on the *CmMYB6*, *CmMYB#7* and *CmbHLH2* promoters. Different lowercase letters above bars represent statistically significant differences at the 0.05 probability level. Error bar indicates the SE of three independent biological replicates.

Since *CmDFR* was the first late biosynthetic genes and it was strongly induced by both *CmMYB6-CmbHLH2* and *CmMYB6-CmbHLH2.1*, the regulatory effects of two bHLHs together with CmMYB6 on *CmDFR* promoter were analyzed. Both CmbHLH2 and CmbHLH2.1 together with CmMYB6 could activate *CmDFR* promoter but in varying strengths, and the fold-induced activation was increased by more than 40 times and 10 times, respectively, compared to Empty vector (Figure 8A). 

Dual-luciferase assays were carried out to test the regulation effects of the MYB-bHLH complex on regulatory genes, including *CmMYB6*, *CmbHLH2* and *CmMYB#7*. The results showed that neither CmMYB6-CmbHLH2 nor CmMYB6-CmbHLH2.1 had regulatory effects on *CmMYB#7* and *CmMYB6* promoter (Figure 8B). However, *CmbHLH2* promoter could be induced by CmMYB6-CmbHLH2 but not CmMYB6-CmbHLH2.1 (Figure 8B), indicating CmMYB6-CmbHLH2 could activate *CmbHLH2* promoter to provide positive feedback regulation.

## 3. Discussion

Flower color alteration can be divided into heritable and temporary alteration. Heritable variation is generally due to gene mutation, such as insertions of transposons or footprints caused by transposon excisions, which can be passed on to the next generation [28,29]. The temporary color change is mainly caused by environmental stimuli, such as high-light conditions, low temperatures, drought, etc., which are regulated by gene expression changes [2,23,30]. Based on transcriptome data, the biosynthetic genes of the anthocyanin pathway were all up-regulated in turning red ‘Jimba’ (Figure 3). Generally, anthocyanin biosynthetic genes are conservatively regulated by the MBW complex, among which MYB is the most specific unit, including activator and inhibitor. Based on differentially expressed genes between white and turning red ‘Jimba’, we screened out an MYB from the S6 subfamily (Figure 4A), whose amino acid sequence shared 97% similarity with CmMYB6, a characterized anthocyanin activator [24]. Here, we confirmed that CmMYB6 is a functional activator in ‘Jimba’ based on the transient over-expression assay, and its trans-activation was bHLH-dependent (Figure 7A). Over-expressed *35S::CmMYB6* could not separately induce anthocyanin in ‘Jimba’ petals (data not shown), indicating the essential role of bHLHs.

Up to now, only CmbHLH2 has been functionally characterized as a co-regulator with CmMYB6 in chrysanthemum. Here, we identified another bHLH named CmbHLH2.1, which is also involved in anthocyanin regulation in chrysanthemum. Transiently over-expressed *CmbHLH2.1* together with *CmMYB6* could induce anthocyanin accumulation in white ‘Jimba’ petals (Figure 7A), and all anthocyanin biosynthetic genes were up-regulated (Figure 7C). Moreover, the expression of *CmMYB#7*, a charactered anthocyanin repressor, was significantly inhibited in both *35S::CmMYB6-CmbHLH2* and *35S::CmMYB6-CmbHLH2.1* transformed petals (Figure 7C), indicating that CmMYB6-CmbHLH2 and CmMYB6-CmbHLH2.1 activate the anthocyanin pathway both by regulating anthocyanin biosynthetic genes and regulatory genes.

According to the dual-luciferase assay, the promoter of *CmbHLH2* could be induced by CmMYB6-CmbHLH2 (Figure 8B), indicating that there is a positive feedback regulation between transcription factors, which is consistent with TT8 in *Arabidopsis* and AN1 in Petunia. They can be activated by MBW complex and then provide a positive-feedback loop to ensure a strong accumulation of anthocyanins in plant organs [8,31]. However, the promoters of *CmMYB#7* and *CmMYB6* were not regulated by the MYB-bHLH complex based on dual-luciferase assays (Figure 8B).

The bHLH family is defined by the bHLH signature domain, which constitutes an N-terminal basic region that binds DNA to the canonical E-box (CANNTG) and a helix-loop-helix (HLH) domain involved in homo- and/or heterodimerization [11,32]. Additionally, anthocyanin-related bHLHs contain a MIR domain for interacting with MYB. CmbHLH2 and CmbHLH2.1 both harbor an MIR domain at the N-terminal (Figure 5B), and the interactions with CmMYB6 were further confirmed by Yeast two-hybrid assays (Figure 6A). Although CmMYB6-CmbHLH2.1 could form a complex and induced anthocyanin accumulation in chrysanthemum, the regulatory effect was far less than CmMYB6-CmbHLH2 (Figure 7C and Figure 8A). The differences between two bHLHs were only 26 amino acids at the C-terminal (Figure 5), indicating C-terminal region of bHLH protein might be closely associated with regulatory activity. It has been verified that C-terminal region of anthocyanin-related bHLHs mediated homo-/heterodimers formation. In maize, Feller et al. showed that R (anthocyanin-related bHLH) contains an ACT-like domain at C-terminal region that can direct the formation of homodimers in vitro and in vivo, and this dimerization domain is necessary for the R regulatory activity. Moreover, Kong et al. showed that R homodimer formation through the ACT-like domain at C-terminal region prevents the bHLH dimeric arrangement that is essential for DNA-binding activity [11,12,13]. Thus, we speculated the different DNA-binding activity between two bHLHs on *CmDFR* promoter might be due to the amino acid variations at the C-terminal region (Figure 6B). 

In chrysanthemum, as far as we know, there are three kinds of splicing variants of *bHLH2* precursor mRNA, including *CmbHLH2*, *CmbHLH2^short^* and *CmbHLH2.1*. *bHLH2^short^* encode a truncated protein with a partial MYB-interaction region, thus failing to regulate anthocyanin biosynthesis as a co-regulator [33]. On this basis, we found another splicing variant, *CmbHLH2.1*, which is a functional anthocyanin activator, but the regulatory effect is less than *CmbHLH2*. These findings suggest alternative splicing may act as a potential approach to modulate anthocyanin biosynthesis in chrysanthemum. It is worth to further investigate whether these splicing variants are regulated by internal or external factors.

## 4. Materials and Methods

### 4.1. Plant Materials

‘Jimba’ chrysanthemum used in this study was cultivated in natural conditions located in Hangzhou, Zhejiang Province (30°150′ N, 120°100′ E). Petals were collected at full-bloom stage of white and turning red ‘Jimba’. Three replicates were collected for each sample and frozen by liquid nitrogen, then stored in −80 °C for future use.

### 4.2. RNA Extraction and cDNA Synthesis

The CTAB method was used for total RNA extraction as described previously [34]. The integrity of the RNA was analyzed by agarose gel electrophoresis. After removing DNA contamination by DNase I (Invitrogen, Waltham, MA, USA; Lithuania), first-strand cDNA was produced by reverse transcriptase (Bio-rad, Hercules, CA, USA).

### 4.3. Anthocyanin Content Analysis

The relative anthocyanin contents of flower petals were determined using the method described previously [35]. Briefly, frozen tissue was ground to a powder and then extracted in 600 μL of methanol acidified with 1% HCl, and kept overnight at 4 °C in the dark. The acidified methanol was diluted to a 60% solution by the addition of 400 μL of water before extraction with an equal volume of chloroform. Total anthocyanins were determined by measuring absorbance at 530 nm and 657 nm of the aqueous phase using a UV-2550 spectrophotometer (SHI-MADZU).

### 4.4. Transcriptome Library Construction, Sequencing and Data Analysis

The library construction, sequencing and data analysis were all conducted by Novogene in Beijing. A total of 1.5 μg of RNA was used for library construction using the NEBNext^®^ Ultra TM RNA Library Prep Kit from Illumina^®^ (NEB, Ipswich, MA, USA). Sequencing libraries were generated using the NEBNext^®^ UltraTM RNA Library Prep Kit from Illumina^®^ (NEB, Ipswich, MA, USA), following the manufacturer’s recommendations, and index codes were added to attribute sequences to each sample. 

The clustering of the index-coded samples was performed on a cBot Cluster Generation System using TruSeq PE Cluster Kit v3-cBot-HS (Illumia) according to the manufacturer’s instructions. After cluster generation, the library preparations were sequenced on an Illumina Hiseq platform and paired-end reads were generated.

Clean data were obtained by removing reads containing adapter, reads containing ploy-N and low-quality reads from raw data. The obtained clean data were spliced with Trinity (v2.6.6) software. Transcript functions were annotated based on the following databases: NR (NCBI non-redundant protein sequences); NT (NCBI non-redundant nucleotide sequences); Pfam (Protein family); KOG/COG (Clusters of Orthologous Groups of proteins); Swiss-Prot (A manually annotated and reviewed protein sequence database); KO (KEGG Ortholog database); GO (Gene Ontology).

### 4.5. Screening of Differentially Expressed Genes and KEGG Enrichment Analysis

The criteria for differentially expressed genes screening are: padj ≤ 0.05, |Log_2_ (fold change)| ≥ 1 and FPKM ≥ 10. KOBA (v2.0.12) software was used for KEGG enrichment analysis of differential genes. The enrichment analysis is based on the principle of hypergeometric distribution, where the differential gene set is a collection of differential genes annotated to the KEGG database, and the background gene set is a collection of all genes annotated to the KEGG database.

### 4.6. Phylogenetic Analysis and Protein Sequence Alignment

Phylogenetic tree was constructed by MEGAX version X with a bootstrap value of 1000 using the neighbor-joining tree method [36]. ClustalX was used for protein sequence alignment with bHLHs, including VvMYC (*Vitis vinifera*, GenBank accession number EU447172), AtbHLH001 (*Arabidopsis thaliana*, NP_001332705.1), AtbHLH002 (*A. thaliana*, NP_001185302.1), AtbHLH042 (*A. thaliana*, NP_192720.2), MrbHLH1 (*Myrica rubra*, JX629461), MrbHLH2 (*M. rubra*, JX629462), MdbHLH3 (*Malus domestica*, HM122458), MdbHLH33 (*M. domestica*, DQ266451), LjTT8 (*Lotus japonicas*, BAH28881), AN1 (*Petunia hybrid*, AF260918), NtAn1a (*Nicotiana tabacum*, AEE99257), NtAN1-like (*N. tomentosiformis*, AEE99260), NtAn1b (*N. tabacum*, AEE99258), AmDELILA (*Antirrhinum majus*, AAA32663), L-c (*Zea mays*, NM001111869) and R-S (*Z. mays*, X15806). Then, GENEDOC was used for comparison of conserved amino acids, indicated by shading in the figures [37,38].

### 4.7. Yeast Hybrid Assays

Yeast one-/two-hybrid assays were carried out to analyze the protein–DNA or protein–protein interaction according to the manufacturers’ instruction (Matchmaker Gold Yeast Two-Hybrid System, Clontech, Mountain View, CA, USA). For Yeast two-hybrid assays, *CmMYB6* and *CmbHLH2*/*CmbHLH2.1* were cloned into GAL4-AD and GAL4-BD vectors, respectively. Then, GAL4-BD was transformed into the Y2H yeast strain, and the appropriate concentrations of aureobasidin A (AbA) inhibiting self-transactivation were tested on SD/−Ura+AbAx medium. The minimum AbA concentration that inhibited the auto-activation of *bHLH2.1* and *bHLH2* bait strain were 200 mg/L and 400 mg/L, respectively. GAL4-AD and GAL4-BD vectors containing target genes were co-transformed into the yeast two-hybrid strain and the interactions were detected on quadruple medium (SD/−Ade/−His/−Leu/−Trp+ X-α-Gal+ AbA). 

For Yeast one-hybrid assays, *CmbHLH2* and *CmbHLH2.1* were cloned into GAL4-AD and the promoter region of *CmDFR* was cloned into the pAbAi bait strain, respectively. The linearization vector of *CmDFR*-pAbAi was transformed into Y1H yeast strain, and then the appropriate concentration of AbA inhibiting self-transactivation was tested on SD/−Leu+AbAx medium. The minimum ABA (Aureobasidin A) concentration to inhibit auto-activation of *CmDFR*-pAbAi was 175 mg/L. Recombined GAL4-AD vectors were transformed into Yeast one-hybrid strain containing *CmDFR*-pAbAi bait strain, and the interactions were tested on SD/−Leu/+AbA medium.

### 4.8. Transient Over-Expressed Assay

Flower petals of white ‘Jimba’ were chosen for transient over-expression analysis of MYB and bHLHs. Full-length CDS of *CmMYB6* and *CmbHLH2/CmbHLH2.1* were cloned into pGreenII 0029 62-SK vector in tandem and then transformed into Agrobacteria strain GV3101 (MP90). GV3101 (MP90) containing *35S::CmMYB6-CmbHLH2.1*, *35S::CmMYB6-CmbHLH2* and empty vector were infiltrated into flower petals with a syringe removed needle. The infiltrated patches were photographed and sampled eight days after infiltration. Each experiment was carried out with more than three independent biological replicates. 

### 4.9. RT-qPCR

Expression of specific genes was quantified by RT-qPCR using fast EvaGreen supermix Kit (Bio-rad, USA) and a CFX96 instrument (Bio-Rad, USA). RT-qPCR primers listed in Supplemental Table S1 were verified by a melting curve and PCR products sequencing. The program was initiated with the preliminary step of 3 min at 95 °C, followed by 40 cycles of 95 °C for 10 s, 60 °C for 30 s and 95 °C for 10 s. A reference gene *CmACT* (GenBank AB770471) was used to calculate the relative expression of genes with formula 2(−ΔCt). No-template reactions were used as negative controls.

### 4.10. Dual-Luciferase Assay

Dual-luciferase assays were carried out as described previously [25]. Full-length TFs and target promoters (−2000 bp region of *CmMYB6*, *CmbHLH2* and −1374 bp *CmMYB#7*) were cloned into pGreenII 0029 62-SK and pGreenII 0800-LUC vectors, respectively, with the primers listed in Table S2. The recombinant plasmids were transformed into Agrobacteria strain GV3101 (PM90). TFs and promoters were mixed (10:1 vol/vol) and then infiltrated into tobacco leaves (Nicotiana benthamiana). Enzyme activities of firefly luciferase (LUC) and renilla luciferase (REN) were tested using the Dual-Luciferase Reporter Assay System (Promega, Madison, WI, USA). Transcriptional regulatory effects were calculated by the ratio of LUC/REN.

## Data Availability

The datasets supporting the conclusions of this article are included within.

## Supplementary Materials

The following are available online at https://www.mdpi.com/article/10.3390/ijms222312769/s1.
