# Supplementary material for: Comparative Transcriptome Analysis Revealed Two Alternative Splicing bHLHs Account for Flower Color Alteration in Chrysanthemum
Source: Int J Mol Sci. 2021 Nov 25;22(23):12769. doi: 10.3390/ijms222312769 (PMC8657904; doi:10.3390/ijms222312769)
Supplement: Supplementary file 1 [file ijms-22-12769-s001.zip › ijms-1472269-supplementary.pdf]

Table S1. Primers used for qRT-PCR

**Gene**	**Forward (5'-3')**	**Reverse(5'-3')**
CmMYB6	ACGGCGAAATAGGGTGGTCATTAG	GGATTGCAATATCATAGTTGGTCCG
CmMYB#7	TACAGGATGCACAAGCTTGTTG	ACATCGTATGAGACAAAGTGTC
CmbHLH2	GGGTTGTTGCTTGACGTGATGAAG	GCTAATTTTCTTCCCCCTAATACC
CmbHLH2.1	ACCGTTCAATCCTGTGTCG	CGTCGTTCAAGCAAGAGC
CmCHS	CAAGGAGGAGAAGATGAGAG	CCGAACCCGAATAAAACAC
CmCHI	GCAGGTGTGAGAGGTATG	GCAACGGAATCGCTTTATC
CmF3H	CACGGGTAATGTTAGGTAGG	TGTAGGTCAAATCGGTCAAG
CmF3'H	AGGCGGATTCATCGTTTC	ACTCTTTGGGCTTATCAGG
CmDFR	TAGTAACAAAGGCGGACAC	GGATTATTCACCAAGTATGCTC
CmANS	GGGCTCCAACTACTCTATG	TCCTAACCTTCTCCTTATTCAC
CmUGT	ATCACAGGGACTATCAACC	TCCACCACCAGACAACTA
CmGST	CCAAAAATGGGTGGTAAAA	TAAGATCCGCAAGGGTGAA

Table S2. Primers used for LUC vector construction

	**Forward (5'-3')**	**Reverse(5'-3')**
CmMYB6	CAGCCCGGGGGATCCGTAATGTTGCATGTATCCAAAC	TGGCGTCTTCCATGGTTTTACTCTTATATCCCTAATTG
CmMYB#7	CAGCCCGGGGGATCCGTATTCCAGATATCATGAGTGG	TGGCGTCTTCCATGGCAGTTGAAAGGATAGGTTAGG
CmbHLH2	CAGCCCGGGGGATCCGGAAGCAAGTAAATCGGAGG	TGGCGTCTTCCATGGCGCTAACCACCTCCTTAGCTTC

Note: Added restriction enzyme sites are underlined
